# Molecular epidemiology and spatiotemporal dynamics of norovirus associated with sporadic acute gastroenteritis during 2013–2017, Zhoushan Islands, China

**DOI:** 10.1371/journal.pone.0200911

**Published:** 2018-07-18

**Authors:** Can Chen, Jian-Bo Yan, Hong-Ling Wang, Peng Li, Ke-Feng Li, Bing Wu, Hui Zhang

**Affiliations:** 1 Department of Public Health, Nanchang University, Nanchang, Jiangxi Province, China; 2 Zhoushan Center for Disease Control and Prevention, Zhoushan, Zhejiang Province, China; 3 Zhejiang Provincial Key Laboratory of Health Risk Factors for Seafood, Zhoushan Center for Disease Control and Prevention, Zhoushan, Zhejiang Province, China; University of Hong Kong, HONG KONG

## Abstract

A total of 1 590 fecal swabs and stool samples from sporadic acute gastroenteritis patients of all ages were collected from January 2013 to March 2018 in the Zhoushan Islands, China, with 99 (6.23%) samples subsequently identified as *human norovirus (HuNoV)* positive. Phylogenetic analysis of partial RdRp and VP1 gene regions identified 10 genotypes of the GII genogroup and 3 genotypes of the GI genogroup. The predominant genotype was GII.P17-GII.17 (42.86%, 33/77), followed by GII.Pe-GII.4_Sydney 2012 (24.68%, 19/77) and GII.P16-GII.2 (12.96%, 10/77). However, the prevailing genotype in the Zhoushan Islands has shifted on three separate occasions. The GII.Pe-GII.4_Sydney_2012 strain was dominant in 2013–2014, the GII.P17-17 strain was dominant in 2015–2016, and the GII.P16-GII.2 strain was dominant in 2017. Divergence analysis showed that the re-emerging GII.P16-GII.2 strains clustered with the Japanese 2010–2012 GII.P16-GII.2 strains, and the time of the most recent common ancestor was estimated to have occurred in 2012 to 2013. The evolutionary rates of the RdRp gene region of the GII.P16 genotype and the VP1 gene region of the GII.2 genotype were 2.64 × 10^−3^ (95% HPD interval, 2.17–3.08 × 10^−3^) and 3.36 × 10^−3^ (95% HPD interval, 2.66–4.04 × 10^−3^) substitutions/site/year, respectively. The migration pattern of the HuNoV GII.2 genotype in China demonstrated that the re-emerging GII.P16-GII.2 strains were first introduced into Hong Kong from Japan, and then spread from Hong Kong to other coastal areas. Our results also showed that the GII.P16-GII.2 strains in the Zhoushan Islands were likely introduced from Jiangsu Province, China, in 2016.

## Introduction

The human norovirus (HuNoV) has been responsible for most sporadic and major outbreaks of non-bacterial gastroenteritis since its first discovery in 1972 [1, 2]. Due to its high infectivity and efficient transmission, HuNoV-associated acute gastroenteritis is an important public health issue worldwide, affecting humans in all age groups [3]. HuNoV is associated with 18% of all gastroenteritis cases and causes over 200 000 deaths in developing countries annually [4].

Belonging to the family *Caliciviridae*, HuNoV has a single-stranded, positive-sense RNA genome of approximately 7.5 kb in length, which contains three open reading frames (ORFs). ORF1 encodes a large non-structural polyprotein, and includes p28, NTPase, p22, VPg, 3C-like protease (3CLpro), and RNA-dependent RNA polymerase (RdRp). ORF2 and ORF3 encode the major structural protein VP1 and minor structural protein VP2, respectively. VP1 consists of a shell (S) domain and a protruding (P) domain. The P domain is responsible for binding to histo-blood group antigens (HBGAs), which function as attachment factors or co-receptors on host cells, and contains significant determinants of antigenicity [5, 6]. The frequent recombination that occurs in ORF1-ORF2 overlap regions and antigenic variation in the P domain likely contribute to the emergence of novel strains, thus enabling immune evasion [7].

Based on sequence differences in the RdRp and VP1 gene regions, HuNoV can be classified into at least seven genogroups (GI–GVII), with GI, GII, and GIV notable for their ability to infect humans [8]. GI and GII are the two major genogroups among HuNoV-associated acute gastroenteritis cases and can be further divided into at least 31 different genotypes [9]. Since 1990, the GII.4 genotype has dominated globally. New GII.4 variants emerge every two to three years and replace the previously dominant GII.4 strains in subsequent seasons. The GII.4_Sydney_2012 strain identified in 2012–2013 is the latest GII.4 variant circulating globally [10]. In the winter of 2014–2015, a new GII.P17-GII.17 strain replaced GII.4, emerging as a major cause of acute gastroenteritis outbreaks in partial regions of Asia [11]. In late 2016, the re-emerging recombinant GII.P16-GII.2 genotype overwhelmed the GII.P17-GII.17 and GII.4 genotypes, with increasing detection in China and Germany [12, 13]. Many countries have since reported GII.P16-GII.2 strains as dominant in HuNoV-associated acute gastroenteritis outbreaks in 2016 and 2017 [14, 15, 16, 17].

China is one of 15 countries with the highest burden of diarrhea disease worldwide, with the HuNoV infection rate increasing from 11.20% in 2007 to 15.30% in 2012 [18]. HuNoV in China is highly diverse [19] and once a new strain or variant emerges, China is one of the main regions where these strains can become an epidemic, including the GII.4_Hunter_2004, GII.4_DenHaag_2006b, GII.4_New Orleans_2009, GII.4_Sydney_2012, GII.P17-GII.17, and latest GII.P16-GII.2 strains [20, 21, 22, 12]. Currently, no licensed norovirus vaccine exists, but candidates vaccines are being tested in clinical trials. Vaccine design is complicated by frequently antigenic variation within the genus, and currently is just targeting most common genotypes [23, 24]. The epidemiology and disease burden of distinct genotypes are also different. Hence, it is necessary to monitor the prevalence and variation of HuNoV strains for development of prevention and control strategies. Sporadic HuNoV-associated gastroenteritis is an important area of public health issue. In this study, we performed continuous molecular surveillance and phylogeographic analysis to illustrate the prevalence, epidemic genotypic diversity, and spatiotemporal dynamics of HuNoV strains in sporadic gastroenteritis cases from 2013 to 2017 in the Zhoushan Islands, China.

## Materials and methods

### Specimen collection

A total of 1 590 fecal swabs and stool samples from acute gastroenteritis patients of all ages were collected between January 2013 and March 2018 by a regional HuNoV gastroenteritis surveillance program conducted at Zhoushan Hospital, Zhejiang, China. All specimens were sent to the Zhoushan Center for Disease Control and Prevention for HuNoV detection. The study was approved by the ethics committee of the Zhoushan Center for Disease Control and Prevention.

### RNA extraction, norovirus detection, amplification, and genetic analysis

Viral RNA was extracted from 10% phosphate-buffered saline specimen suspensions (200 μl) using a Qiagen Viral RNA Mini Kit (Qiagen, Hilden, Germany) according to the manufacturer’s protocols. The RNA was then subjected to real-time fluorescent quantitative polymerase chain reaction (RT-qPCR) for HuNoV detection using genogroup-specific primers and probes (JJV1F/JJV1R/JJV1P and JJV2F/COG2R/RING2-TP) as described previously [25]. HuNoV-positive RNA products were subjected to reverse transcription PCR (RT-PCR) for amplification partial RdRp and VP1 gene regions of HuNoV GI and GII using primers MON432/G1SKR (M87661 nucleotide position: 5 093–5 636 bp) and MON432/G2SKR (X86557 nucleotide position: 4 820–5 389 bp) according to protocols described previously [26, 27]. All PCR products were purified and sequenced by Sangon Biotech (Shanghai, China). The obtained sequences were designated by nomenclature [28] and the online NoV typing tool v.1.0 (http://www.rivm.nl/mpf/norovirus/typingtool) was used to determine genotypes for the obtained HuNoV strain sequences. Sequence alignment was performed using ClustalW and phylogenetic trees were constructed by neighbor-joining (NJ) with MEGA v.7.0 software [29]. SPSS v.20.0 was used to test annual infection rate by *χ*^*2*^ analysis.

### Amplification of VP1 and RdRp gene regions and complete genomes of GII.P16-GII.2 strains

Ten samples were identified as GII.P16-GII.2 strains from the Zhoushan Islands in 2017. We applied primers RdRp-3875F (5'-CAAAAATGGACATACGCACAG-3') / RdRp-5259R (5'-CCTGGACAAAATTTGCTCTAA-3') and VP1-5025F (5'-TGAG GTTTTCTGACTTGAGCA-3')/ VP1-6731R (5'-CTACAAAAGCTCCAGCCATTA -3') to amplify the nearly complete VP1 (KY421122 nucleotide position: 5 025–6 731 bp) and partial RdRp (KY421122 nucleotide position: 3 875–5 259 bp) gene regions in a 25-μl reaction volume using a Qiagen One-Step RT-PCR kit (Qiagen, Hilden, Germany) according to the manufacturer’s instructions. RT-PCR was performed at 42°C for 30 min and 95°C for 15 min, followed by 35 cycles at 95°C for 1 min, 58°C for 1 min, 72°C for 2 min, and a final extension at 72°C for 7 min. The PCR products were purified and sequenced by Sangon Biotech (Shanghai, China). We obtained 2.7 kb genomic fragments from eight HuNoV strains. The complete GII.P16-GII.2 strain genome was determined by Sangon Biotech (Shanghai, China) using Sanger dideoxy sequencing. The amplification method was accroding to described above and primers sequences which used for amplifying complete GII.P16-GII.2 strain genome was uploaded in supporting information S3 Table. All sequences are available in GenBank under accession numbers MH321817 to MH321825.

### Spatiotemporal of GII.P16-GII.2 in the Zhoushan Islands

To examine the spatiotemporal dynamics of the re-emerging GII.P16-GII.2 genotype of HuNoV in the Zhoushan Islands, we collected 123 sequences of the GII.P16 RdRp gene region (> 1 100 bp) (S1 Table) and 269 sequences of the GII.2 VP1 gene region (> 1 550 bp) (S2 Table) of HuNoV strains from the GenBank nucleotide database of the National Center for Biotechnology Information (NCBI) (https://www.ncbi.nlm.nih.gov) and eight sequences of the GII.P16 RdRp and GII.2 VP1 gene regions of HuNoV strains from our study in March 2018. The VP1 gene sequences of the GII.2 strains were used to generate Bayesian phylogeographic trees, whereas the RdRp gene sequences were employed to create a time-scale evolutionary tree. Analysis was implemented in BEAST v.1.8.2 using the Bayesian Markov Chain Monte Carlo (MCMC) [30] method and Bayesian stochastic search variable selection (BSSVS) model [31].

We first selected the best-fit nucleotide substitution model using jModelTest2 [32]. Appropriate clock and tree models were then determined by path-sampling/stepping stone-sampling (PS/SS) in BEAST v.1.8.2, with MCMC chain lengths of 100 million steps and sampling every 10 000 steps. After omission of the first 10% of the chain, the convergence of continuous parameters was evaluated by effective sample size (ESS) using Tracer v.1.6 (http://tree.bio.ed.ac.uk/software/tracer/). Maximum clade credibility (MCC) trees were then constructed after first 10% burn-in of the trees using TreeAnnotator in BEAST v.1.8.2 [30] and visualized using FigTree v.1.4.3 (http://tree.bio.ed.ac.uk/software/figtree/). We also estimated the evolutionary rates of the VP1 and RdRp gene regions using appropriate models, as described above. To visualize the temporal transmission routes for the HuNoV GII.2 genotype, MCC trees were converted into a keyhole markup language (KML) file using SPREAD v.1.0.7 [33], which is suitable for visualization with Google Earth (http://earth.google.com). Bayes factor (BF) tests [31] were conducted for the HuNoV GII.2 genotype using SPREAD v.1.0.7 software with the BSSVS model to provide statistical support for the significant transmission routes between discrete locations, a more likely migration may exist between two locations with high BF value. Thus, we considered a migration route with BF > 1000 to be a significant transmission route.

## Results

### Prevalence of HuNoV-associated sporadic acute gastroenteritis

Between January 2013 and March 2018, 1 590 fecal swabs and stool samples collected from HuNoV-associated patients of all ages were screened for HuNoV. In total, 99 (6.23%) specimens were identified as HuNoV-positive (Table 1), among which 94 (94.95%) belonged to the GII genogroup, 3 (3.03%) belonged to the GI genogroup, and 2 showed GI and GII co-infection (2.02%). The infection rates were significantly different by year (*χ*^*2*^ = 16.13, *P* < 0.05), and the increasing infection rate trend exhibited significant differences by year (*χ*^*2*^_*trend*_ = 8.77, *P* < 0.05). The infection rate has increased since 2014, the infection rates significantly different between 2014 from 2015 (*χ*^*2*^ = 8.063, *P* < 0.01^a^).

**Table 1:** HuNoV infection rates of sporadic acute gastroenteritis in the Zhoushan Islands between 2013 and 2017.

Year	Sample	Positive sample	Infection rate (%)	Obtained sequences
2013	420	15	3.56	8
2014	425	20	4.71	12
2015	194	21	10.82	18
2016	182	14	7.69	11
2017	369	29	7.80	29
Total	1 590	99	6.23	78

^a^Significance level *α* was adjusted by bonferroni method.

### Genetic diversity and shift in epidemic genotype

**Fig 1:**
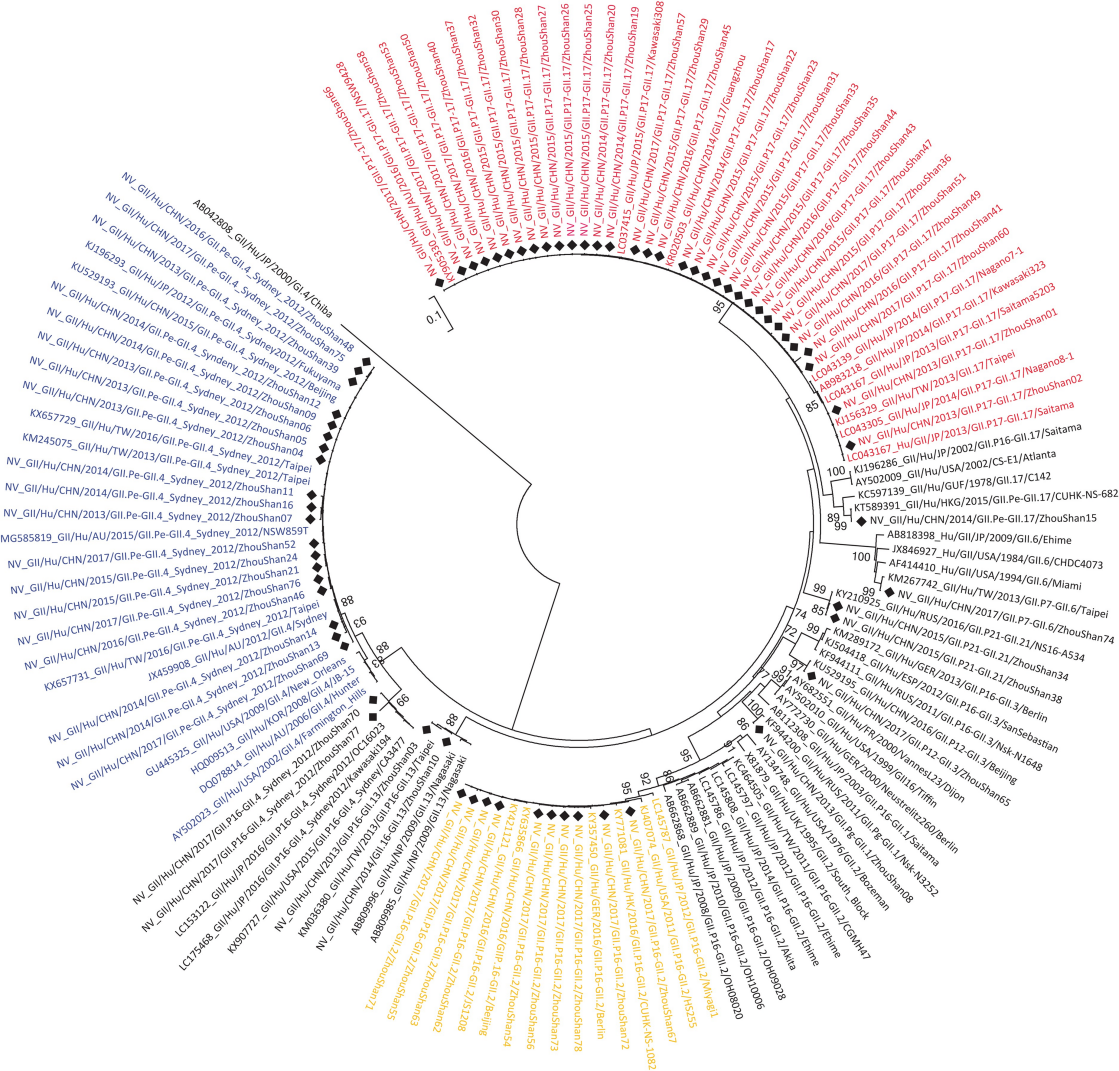
Phylogenetic analyses of 77 sequences of HuNoV strains based on partial RdRp and VP1 gene regions. Trees were generated using the Neighbor-Joining (NJ) method and genetic distances were calculated using the Kimura 2-parameter + Gamma substitution model employed in MEGA v.7.0. Bootstrap values (>70%) are shown at the corresponding branches. HuNoV strains isolated in this study are marked with solid black diamonds. Blue represents GII.Pe-GII.4_Sydney_2012, red represents GII.P17-GII.17, yellow represents GII.P16-GII.2.

A total of 78 HuNoV strains from the 99 positive samples were further identified by sequencing the partial RdRp and VP1 gene regions. Ten genotypes of the GII genogroup and three genotypes of the GI genogroup were confirmed using the NoV genotyping tool v.1.0 (Table 2). From 2013 to 2017, the main genotype was the GII.P17-GII.17 (42.86%, 33/77) strain, followed by the GII.Pe-GII.4_Sydney_2012 (24.68%, 19/77) and GII.P16-GII.2 (12.96%, 10/77) strains (Table 3). The main genotype responsible for HuNoV-associated sporadic acute gastroenteritis epidemics has changed on three separate occasions. The GII.Pe-GII.4_Sydney_2012 strain dominated during 2013–2014, and all GII.4_Sydney_2012 strains were clustered closely with the JX459908/Sydney/2012/AU strain, sharing 99.2%–99.7% identity according to phylogenetic analysis. Replacing the GII.Pe-GII.4_Sydney_2012 strain, the new GII.17 strains emerged in the winters of 2014–2015 and were clustered closely with the KR020503/CHN/2014/Guangzhou strains, sharing 99.7%–100% identity. In March 2017, the GII.P16-GII.2 strain was first detected, after which it become the dominant genotype in the Zhoushan Islands. All re-emerging GII.P16-GII.2 strains shared 99.5%–100% identity with the KY421121/CHN/2016/Jiangsu strain (Fig 1). The Blast search results of the complete GII.P16-GII.2 strain genome from the Zhoushan Islands also showed 99% identity with the KY421121/CHN/2016/Jiangsu strain. The age distribution of HuNoV infection according to the three dominant genotype have showed significantly different (*χ*^*2*^ = 14.311, *P* < 0.05) (Table 4). The further pairwise *χ*^*2*^ test found that significantly different of age distribution between GII.P17-GII.17 and GII.Pe-GII.4_Sydney_2012 (*χ*^*2*^ = 14.304, *P* < 0.017^a^)

**Table 2:** Distribution of genotypes of HuNoV strains in the Zhoushan Islands from 2013 to 2017.

Year	Genotype	Sequence number
2013–2014	GII.Pe-GII.4_Sydney_2012	10
	GII.P17-GII.17	5
	GII.P16-GII.13	2
	GIIPe-GII.17	1
	GII.Pg-GII.1	1
	GI.Pb-GI.6	1
2015	GII.P17-GII.17	14
	GII.Pe-GII.4_Sydney_2012	2
	GII.P21-GII.21	2
2016	GII.P17-GII.17	7
	GII.Pe-GII.4_Sydney_2012	3
	NA-GII.3	1
2017	GII.P16-GII.2	10
	GII.P17-GII.17	7
	GII.Pe-GII.4_Sydney_2012	4
	GII.P16-GII.4_Sydney_2012	2
	GII.P12-GII.3	1
	GII.P7-GII.6	1
	GI.Pc-GI.5	3
	GI.P2-GI.2	1
Total		78

**Table 3:** Genotypes of HuNoV strains identified in the Zhoushan Islands from 2013 to 2017.

Genotype	Sequence number (sequence proportion)
GII.P17-GII.17	33 (42.86%)
GII.Pe-GII.4_Sydney_2012	19 (24.68%)
GII.P16-GII.2	10 (12.96%)
GII.P16-GII.13	2 (2.60%)
GII.P21-GII.21	2 (2.60%)
GII.P16-GII.4_Sydney_2012	2 (2.60%)
GII.P12-GII.3	1 (1.30%)
GII.P7-GII.6	1 (1.30%)
GIIPe-GII.17	1 (1.30%)
GII.Pg-GII.1	1 (1.30%)
GI.Pc-GI.5	3 (3.90%)
GI.P2-GI.2	1 (1.30%)
GI.Pb-GI.6	1 (1.30%)

**Table 4:** Age distribution of HuNoV infection according to the three dominant genotype.

Age	GII.Pe-GII.4_Sydney_2012	GII.P17-GII.17	GII.P16-GII.2	total
0–14	4	0	1	5
15–40	5	20	7	32
41–60	5	9	0	14
>60	5	4	2	11
total	19	33	10	62

^a^ Significance level *α* adjusted by bonferroni method

### Time-scaled phylogenetic analyses of GII.P16 RdRp

**Fig 2:**
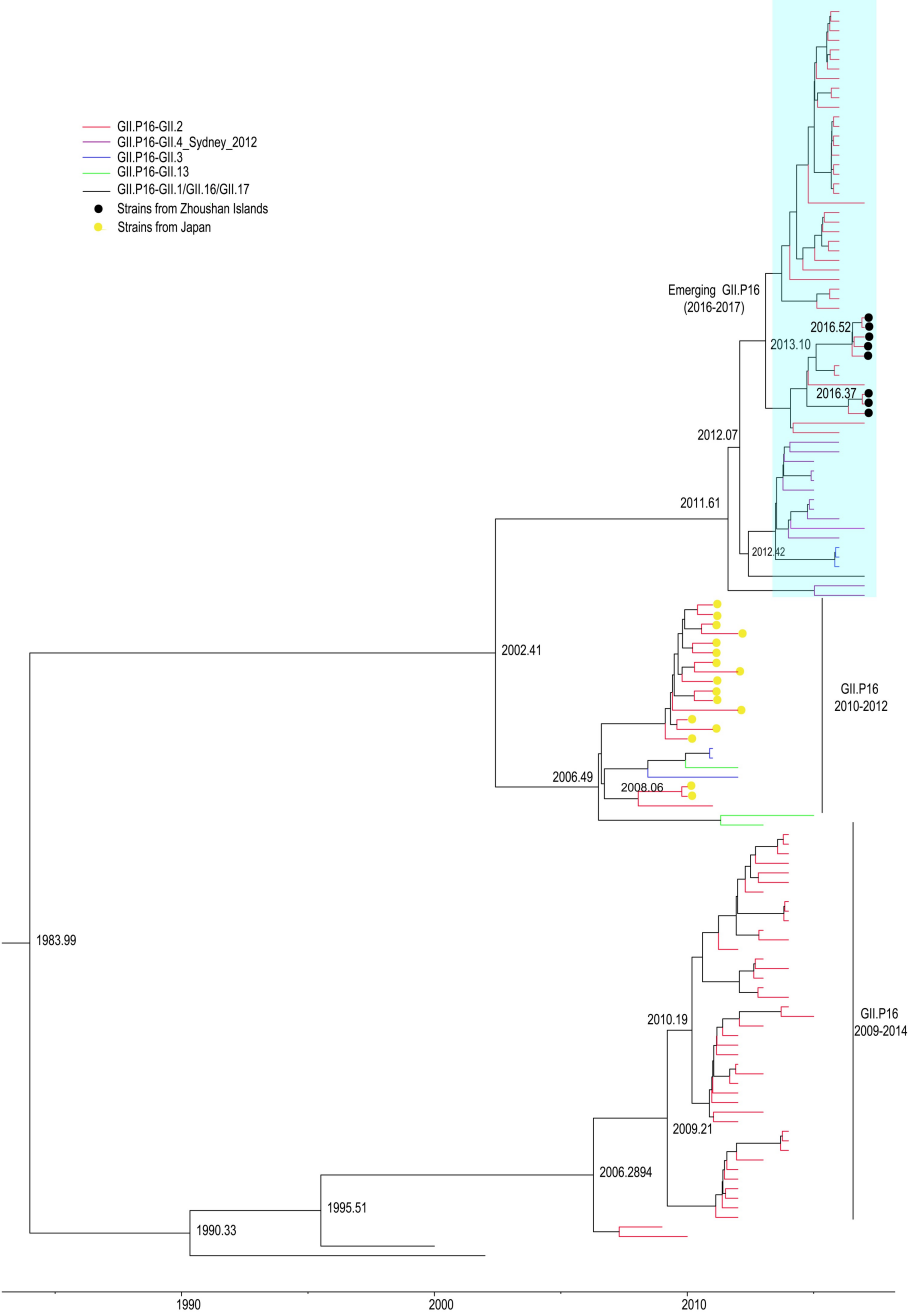
Time-scaled phylogenetic tree of GII.P16 RdRp gene region. Time-scaled phylogenetic tree of 131 sequences of the RdRp gene region of HuNoV GII.P16 genotype strains (1 168 bp) constructed by the Bayesian MCMC method. Branches are colored according to the distinct HuNoV genotype. HuNoV strains isolated in this study are marked with solid black diamonds.

A time-scaled phylogenetic tree of the RdRp gene region of the GII.P16 genotype was generated using the HKY+G nucleotide substitution, strict clock, and constant size coalescent models. The MCC tree showed that the GII.P16 RdRp gene region of the re-emerging GII.P16-GII.2 strains in 2016–2017 diverged from the GII.P16-GII.4_Sydney2012/GII.3 strain (2015–2017), and all re-emerging GII.P16 strains appearing in 2012–2013 clustered with the previous Japanese 2010–2012 GII.P16-GII.2 strains. (Fig 2). Furthermore, the evolutionary rate of the RdRp gene region of the HuNoV Gll.P16 strains was estimated to be 2.64 × 10^−3^ substitutions/site/year (95% HPD interval, 2.17–3.08 × 10^−3^ substitutions/site/year).

### Spatiotemporal dynamics of GII.2 in Zhoushan Islands

**Fig 3:**
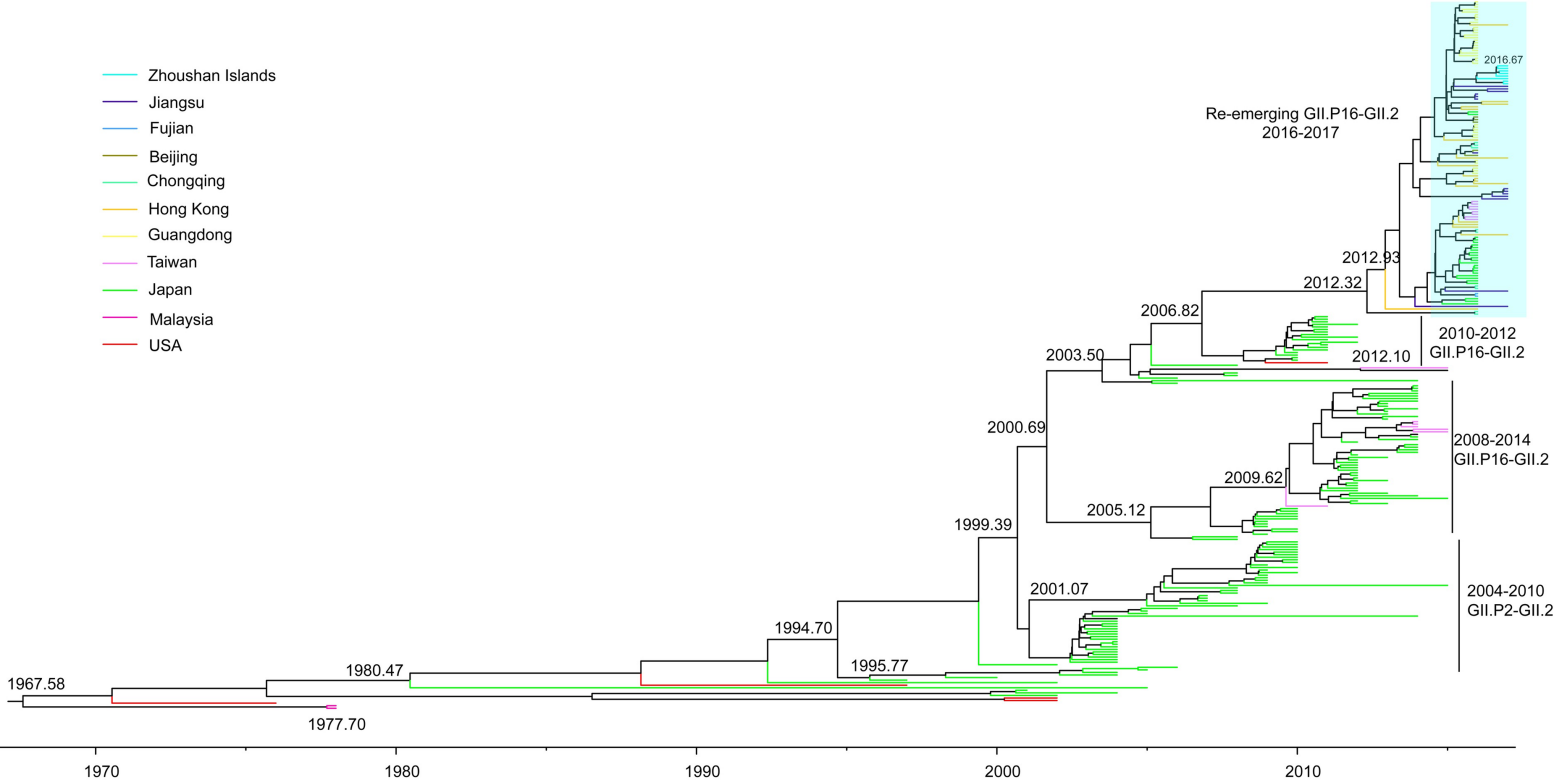
Phylogeographic tree of the VP1 gene of HuNoV GII.2 genotype. Bayesian phylogeographic tree of 277 sequences of the VP1 gene region of HuNoV GII.2 genotype strains (1 555 bp) constructed by Bayesian MCMC method and BSSVS model. Branches are colored according to location state of the descendent nodes.

To understand the dispersal of the GII.P16-GII.2 genotype across natural time scales, 277 sequences of the VP1 gene region of the HuNoV GII.2 genotype, including sequences from the present study, were collected to generate Bayesian phylogeographic trees and estimate divergence time and spatial information for the re-emerging GII.P16-GII.2 strains using the GTR+G+I nucleotide substitution, uncorrelated exponential derivation clock, and constant size coalescent models. Divergence analysis showed that the 2016–2017 re-emerging GII.P16-GII.2 strains appeared in 2012–2013 and clustered with the previous Japanese 2010–2012 GII.P16-GII.2 strains. The Zhoushan Islands strains appeared in 2016 and diverged from Jiangsu strains (KY421126-KY421128) (Fig 3). The evolutionary rate of the VP1 gene of the HuNoV Gll.2 strains was estimated to be 3.36 × 10^−3^ substitutions/site/year (95% HPD interval, 2.66–4.04 × 10^−3^ substitutions/site/year).

The spatial reconstruction of the GII.2 genotype indicated that the virus may have originated in the USA in 1967–1968. By 1977, the virus had spread to Malaysia, and then continued to disperse, reaching Japan in 1980. Over the following several years, the GII.2 genotype accelerated in variation and showed signs of transmission. The earliest GII.2 genotype spread into China in 2009 from Japan to Taiwan, The first re-emerging GII.2 genotype spread into China in 2012–2013, from Japan to Hong Kong. Since then, diffusion of the new GII.2 genotype has intensified in China, from Hong Kong to the coastal regions, including Guangdong, Jiangsu, Beijing, and Fujian, as well as southwest into Chongqing (Fig 3). The strains detected in the Zhoushan Islands were introduced from Jiangsu in 2016.

To identify statistically significant transmission routes between discrete locations, BF tests were conducted for the most significant non-zero rates, with a BF cutoff of 1 000 used to define a significant transmission route. Six significant GII.2 genotype transmission routes were identified. Three of the migration links were related to Japan and Hong Kong, respectively. In China, Taiwan and Hong Kong were closely related to Japan, whereas Guangdong, Jiangsu, and Chongqing were closely related to Hong Kong (Table 5).

**Table 5:** Bayesian factor (BF) tests for significant non-zero dispersion routes of HuNoV GII.2 genotype.

Dispersion route	Bayesian factor value
Hong Kong and Guangdong	3 7287.19
Hong Kong and Jiangsu	9 318.69
Hong Kong and Chongqing	7 454.12
Japan and Hong Kong	3 724.99
Japan and Taiwan	1 198.80
USA and Japan	1 161.21

## Discussion

The present study illustrated the prevalence, epidemic genotypic diversity, and spatiotemporal dynamics of the HuNoV GII.P16-GII.2 genotype strains in the Zhoushan Islands of China from 2013 to 2017 through a five-year continuous surveillance of sporadic acute gastroenteritis patients. The HuNoV infection rate in the Zhoushan Islands (6.23%) was below the average level found in China [18]. As observed for other infectious diseases, such as hand-foot-and-mouth disease, which also exhibits low incidence in the region [34]. This may be due to the lower number of residents in the Zhoushan Islands as well as its geographical segregation from mainland China. The limited influx of people from different regions across China likely restricts the transmission and opportunities of HuNoV infection. However, the *χ*^*2*^_*trend*_ test showed an increasing trend, The infection rate has increased since 2014, the infection rates significantly different between 2014 from 2015. These may be due to in the 2015, a new GII.P17-GII.17 strain emerged as a predominant HuNoV strain. The new GII.17 variant showed highly capability of immune-escaped [35] and a wide binding spectrum to HBGAs [36]. Furthermore, with the development of economy and the convenience of transportation, the population flow and economic trade in Zhoushan Island are becoming more and more frequent.

The HuNoV strains isolated from the Zhoushan Islands showed high diversity and contained 10 genotypes from the GII genogroup and 3 genotypes from the GI genogroup. Significant shifts in predominant HuNoV genotype among sporadic acute gastroenteritis in the Zhoushan Islands were identified. During 2013–2017, the epidemic genotype of HuNoV shifted three times, with the GII.Pe-GII.4_Sydney_2012 strain dominating in 2013–2014, GII.P17-17 strain dominating in 2015–2016, and GII.P16-GII.2 strain dominating in 2017. This trend indicated that the Zhoushan Islands are susceptible to epidemics from new emerging strains. The GII.P16-GII.2 strain was first identified in March 2017 in the Zhoushan Islands, and the GII.P16-GII.2 strain from Zhoushan Islands appeared in 2016 and diverged from the Jiangsu that is slightly later than its epidemic in other regions of China. The Zhoushan Islands are located far from the Chinese mainland, which likely influences the lag in the detection of new variants. The factors that contribute to the prevalence of re-emerging GII.P16-GII.2 may differ from those that contribute to the GII.4 variants and new GII.P17-GII.17 genotype. For the evolution of the GII.4 and GII.17 genotypes, the main driving force was accumulated mutations in the P domain, especially in the antigenic epitopes and HBGA binding interface. That enabled new variant escaped herd immunity and expanded the binding spectrum of HBGA [37, 38]. According to a previous study, however, the antigenicity and HBGA binding profile cannot explain the sudden predominance of re-emerging GII.P16-GII.2 [39]. There are five mutations in the RdRp gene region of the re-emerging GII.P16-GII.2 genotype, several are close to the site that influences polymerase function and viral transmission which reported in the novel GII.P16-GII.4_Sydney_2012 strain [40]. The new RdRp gene region of the GII.P16 genotype may play an important role in the re-emerged GII.P16-GII.2 genotype. There were significantly different of age distribution between GII.P17-GII.17 and GII.Pe-GII.4_Sydney_2012, This results was supported by the previously study [41]. However, no significantly different of age distribution were found between GII.Pe-GII.4_Sydney_2012 and GII.P16-GII.2, GII.P17-GII.17 and GII.P16-GII.2, which may be due to limited number of cases in our study. The GII.Pe-GII.4_Sydney_2012 infected was occured among all age group, but GII.P17-GII.17 (60.61%) and GII.P16-GII.2 (70%) seemed more likely to infect people aged 15–40 years. This results showed that epidemiology of three dominant may be different which need to be further studied especially the re-emerging GII.P16-GII.2. Considering the high genetic diversity, high rate of evolution, and complex epidemiology mechanism of the HuNoV strains, continuous surveillance is required to monitor genotypes and the emergence of new strains.

Divergence analyses of the RdRp and VP1 gene regions of the re-emerging GII.P16-GII.2 genotype showed that the re-emerging GII.P16-GII.2 strains clustered with the 2010–2012 Japanese GII.P16-GII.2 strains. Furthermore, the time of the most recent common ancestor of the re-emerging GII.P16-GII.2 strains appeared during 2012–2013, as supported by previous results [39, 42]. The evolutionary rates of the GII.P16 (2.17–3.08 × 10^−3^ substitutions/site/year) and GII.2 (2.66–4.04 × 10^−3^ substitutions/site/year) genotypes were lower than that of the GII.4 genotype (4.4–7.4 × 10^−3^ substitutions/site/year), but higher than that of the GII.17 genotype (0.79–2.65 × 10^−3^ substitutions/site/year) [43, 44]. The phylogeographic tree showed that the GII.2 strains likely originated from the USA and the novel GII.P16-GII.2 strains emerged from Japan. Migration patterns of the HuNoV GII.2 genotype in China showed that the re-emerging GII.2 strains were first introduced into Hong Kong from Japan in 2012–2013, and then spread from Hong Kong to Guangdong and several other regions in China, mainly in coastal areas. These transmission trends were supported by the high BF values. These migration patterns have also been observed for the new GII.17 genotype prevalence [45]. Earlier research suggested that Guangdong and Hong Kong are the epicenters for HuNoV strains in China, with the overall migration patterns of the HuNoV strains reported from south to north along coastal regions [46]. One plausible speculation for the migration patterns of HuNoV strains in China is via HuNoV-contaminated seafood [47], especially domestic trade of contaminated oysters [48]. A molecular epidemiological study of oyster-related HuNoV strains showed that >80% of HuNoV genotypes were detected in oyster-related outbreaks or oyster samples, and once a new HuNoV genotype or variant emerged from the human host, the consumption of raw oysters could spread the strain [49]. The Zhoushan Islands are one of the largest seafood trade markets in China, and residents often eat raw shellfish [50]. In our study, from 80 cases of HuNoV infection in which the suspicious food was recorded, 37.5% (30/80) was reported to be seafood. This may explain why the Zhoushan Islands are susceptible to newly emerging strain epidemics.

## Conclusions

The HuNoV infection rate in the Zhoushan Islands was below the average level found in China but showed an increasing trend. The HuNoV in Zhoushan Islands is highly diverse and Zhoushan Islands are susceptible to epidemics from new emerging strains. Continuous surveillance to monitor genotypes and the emergence of new strains for development of prevention and control strategies is necessary.

## Supporting information

S1 Table Strains of GII.P16 RdRp used in this study. (DOC)

S2 Table Strains of GII.2 VP1 used in this study. (DOC)

S3 Table The primers sequences used for amplifying complete GII.P16-GII.2 strain genome. (DOC)
