# Supplementary material for: Molecular epidemiology and spatiotemporal dynamics of norovirus associated with sporadic acute gastroenteritis during 2013–2017, Zhoushan Islands, China
Source: PLoS One. 2018 Jul 18;13(7):e0200911. doi: 10.1371/journal.pone.0200911 (PMC6051660; doi:10.1371/journal.pone.0200911)
Supplement: S1 Table — (DOC) [file pone.0200911.s001.doc]

Strains of GII.P16 RdRp used in this study

| accession numbers | sequences names |
| --- | --- |
| MH321817 | Hu/GII/2017/GII.P16-GII.2/ZhouShan |
| MH321818 | Hu/GII/2017/GII.P16-GII.2/ZhouShan |
| MH321819 | Hu/GII/2017/GII.P16-GII.2/ZhouShan |
| MH321820 | Hu/GII/2017/GII.P16-GII.2/ZhouShan |
| MH321821 | Hu/GII/2017/GII.P16-GII.2/ZhouShan |
| MH321822 | Hu/GII/2017/GII.P16-GII.2/ZhouShan |
| MH321823 | Hu/GII/2017/GII.P16-GII.2/ZhouShan |
| MH321824 | Hu/GII/2017/GII.P16-GII.2/ZhouShan |
| AY772730 | Hu/GII/2000/Neustrelitz260/GER |
| KY457726 | Hu/GII/2016/GII.2/TW |
| KY887597 | Hu/GII/2016/GII.P16-GII.3/UK |
| KY887598 | Hu/GII/2016/GII.P16-GII.3/UK |
| KY887599 | Hu/GII/2016/GII.P16-GII.4_Sydney2012/UK |
| KY887600 | Hu/GII/2016/GII.P16-GII.4_Sydney2012/UK |
| KY887601 | Hu/GII/2016/GII.P16-GII.4_Sydney2012/UK |
| KY887602 | Hu/GII/2015/GII.P16-GII.4_Sydney2012/UK |
| KY887603 | Hu/GII/2015/GII.P16-GII.4_Sydney2012/UK |
| KY887604 | Hu/GII/2015/GII.P16-GII.4_Sydney2012/UK |
| KY887605 | Hu/GII/2015/GII.P16-GII.4_Sydney2012/UK |
| KY887606 | Hu/GII/2016/GII.P16-GII.3/UK |
| KY947550 | Hu/GII/2015/GII.P16-GII.4_Sydney2012/USA |
| LC145786 | Hu/GII/2012/GII.P16-GII.2/JP |
| LC145787 | Hu/GII/2012/GII.P16-GII.2/JP |
| LC145788 | Hu/GII/2012/GII.P16-GII.2/JP |
| LC145789 | Hu/GII/2012/GII.P16-GII.2/JP |
| LC145790 | Hu/GII/2012/GII.P16-GII.2/JP |
| LC145791 | Hu/GII/2012/GII.P16-GII.2/JP |
| LC145792 | Hu/GII/2012/GII.P16-GII.2/JP |
| LC145793 | Hu/GII/2012/GII.P16-GII.2/JP |
| LC145794 | Hu/GII/2012/GII.P16-GII.2/JP |
| LC145795 | Hu/GII/2012/GII.P16-GII.2/JP |
| LC145796 | Hu/GII/2012/GII.P16-GII.2/JP |
| LC145797 | Hu/GII/2012/GII.P16-GII.2/JP |
| LC145798 | Hu/GII/2014/GII.P16-GII.2/JP |
| LC145799 | Hu/GII/2014/GII.P16-GII.2/JP |
| LC145800 | Hu/GII/2014/GII.P16-GII.2/JP |
| LC145801 | Hu/GII/2014/GII.P16-GII.2/JP |
| LC145802 | Hu/GII/2014/GII.P16-GII.2/JP |
| LC145803 | Hu/GII/2014/GII.P16-GII.2/JP |
| LC145805 | Hu/GII/2014/GII.P16-GII.2/JP |
| LC145806 | Hu/GII/2014/GII.P16-GII.2/JP |
| LC145807 | Hu/GII/2014/GII.P16-GII.2/JP |
| LC145808 | Hu/GII/2014/GII.P16-GII.2/JP |
| LC175468 | Hu/GII/2016/GII.P16-GII.4_Sydney2012/JP |
| LC209431 | Hu/GII/2013/GII.P16-GII.2/JP |
| LC209432 | Hu/GII/2012/GII.P16-GII.2/JP |
| LC209433 | Hu/GII/2012/GII.P16-GII.2/JP |
| LC209434 | Hu/GII/2014/GII.P16-GII.2/JP |
| LC209441 | Hu/GII/2014/GII.P16-GII.2/JP |
| LC209442 | Hu/GII/2013/GII.P16-GII.2/JP |
| LC209443 | Hu/GII/2013/GII.P16-GII.2/JP |
| LC209444 | Hu/GII/2013/GII.P16-GII.2/JP |
| LC209445 | Hu/GII/2012/GII.P16-GII.2/JP |
| LC209446 | Hu/GII/2012/GII.P16-GII.2/JP |
| LC209447 | Hu/GII/2011/GII.P16-GII.2/JP |
| LC209448 | Hu/GII/2011/GII.P16-GII.2/JP |
| LC209449 | Hu/GII/2011/GII.P16-GII.2/JP |
| LC209450 | Hu/GII/2014/GII.P16-GII.2/JP |
| LC209451 | Hu/GII/2011/GII.P16-GII.2/JP |
| LC209452 | Hu/GII/2011/GII.P16-GII.2/JP |
| LC209453 | Hu/GII/2011/GII.P16-GII.2/JP |
| LC209454 | Hu/GII/2010/GII.P16-GII.2/JP |
| LC209455 | Hu/GII/2013/GII.P16-GII.2/JP |
| LC209456 | Hu/GII/2013/GII.P16-GII.2/JP |
| LC209458 | Hu/GII/2014/GII.P16-GII.2/JP |
| LC209459 | Hu/GII/2010/GII.P16-GII.2/JP |
| LC209460 | Hu/GII/2010/GII.P16-GII.2/JP |
| LC209461 | Hu/GII/2009/GII.P16-GII.2/JP |
| LC209466 | Hu/GII/2012/GII.P16-GII.2/JP |
| LC209467 | Hu/GII/2011/GII.P16-GII.2/JP |
| LC209468 | Hu/GII/2011/GII.P16-GII.2/JP |
| LC209470 | Hu/GII/2014/GII.P16-GII.2/JP |
| LC209471 | Hu/GII/2011/GII.P16-GII.2/JP |
| LC209475 | Hu/GII/2013/GII.P16-GII.2/JP |
| LC209476 | Hu/GII/2013/GII.P16-GII.2/JP |
| LC209477 | Hu/GII/2013/GII.P16-GII.2/JP |
| LC209478 | Hu/GII/2012/GII.P16-GII.2/JP |
| LC209479 | Hu/GII/2011/GII.P16-GII.2/JP |
| LC209480 | Hu/GII/2010/GII.P16-GII.2/JP |
| LC209481 | Hu/GII/2010/GII.P16-GII.2/JP |
| LC213885 | Hu/GII/2015/GII.P16-GII.2/JP |
| LC213886 | Hu/GII/2016/GII.P16-GII.2/JP |
| LC213887 | Hu/GII/2016/GII.P16-GII.2/JP |
| LC213888 | Hu/GII/2016/GII.P16-GII.2/JP |
| LC213889 | Hu/GII/2016/GII.P16-GII.2/JP |
| LC213890 | Hu/GII/2016/GII.P16-GII.2/JP |
| LC213891 | Hu/GII/2016/GII.P16-GII.2/JP |
| LC213892 | Hu/GII/2016/GII.P16-GII.2/JP |
| LC213893 | Hu/GII/2016/GII.P16-GII.2/JP |
| LC213894 | Hu/GII/2016/GII.P16-GII.2/JP |
| LC213895 | Hu/GII/2016/GII.P16-GII.2/JP |
| LC213896 | Hu/GII/2016/GII.P16-GII.2/JP |
| LC213897 | Hu/GII/2016/GII.P16-GII.2/JP |
| LC213898 | Hu/GII/2016/GII.P16-GII.2/JP |
| LC213899 | Hu/GII/2016/GII.P16-GII.2/JP |
| LC213900 | Hu/GII/2016/GII.P16-GII.2/JP |
| LC213901 | Hu/GII/2016/GII.P16-GII.2/JP |
| LC215413 | Hu/GII/2016/GII.P16-GII.2/JP |
| LC215414 | Hu/GII/2016/GII.P16-GII.2/JP |
| LC215415 | Hu/GII/2016/GII.P16-GII.2/JP |
| LC228948 | Hu/GII/2014/GII.P16-GII.2/JP |
| MG002630 | Hu/GII/2017/GII.P16-GII.4_Sydney2012/AU |
| MG002631 | Hu/GII/2017/GII.P16-GII.4_Sydney2012/AU |
| MG002633 | Hu/GII/2017/GII.P16-GII.4_Sydney2012/AU |
| KF895841 | Hu/GII/2012/GII.P16-GII.3/RUS |
| KF944110 | Hu/GII/2011/GII.P16-GII.3/RUS |
| KF944111 | Hu/GII/2011/GII.P16-GII.3/RUS |
| KM036380 | Hu/GII/2013/GII.P16-GII.13/TW |
| KT779557 | Hu/GII/2012/GII.P16-GII.13/RUS |
| KY457727 | Hu/GII/2016/GII.P16-GII.2/TW |
| KY457729 | Hu/GII/2016/GII.P16-GII.2/TW |
| KY457731 | Hu/GII/2016/GII.P16-GII.2/TW |
| KY457734 | Hu/GII/2016/GII.P16-GII.2/TW |
| KY457735 | Hu/GII/2016/GII.P16-GII.2/TW |
| KJ407074 | Hu/GII/2011/GII.P16-GII.2/USA |
| KJ196286 | Hu/GII/2002/GII.P16-GII.17/JP |
| KX907727 | Hu/GII/2015/GII.P16-GII.4_Sydney/USA |
| LC279234 | Hu/GII/2016/GII.P16-GII.2/JP |
| LC279235 | Hu/GII/2016/GII.P16-GII.2/JP |
| LC279236 | Hu/GII/2016/GII.P16-GII.2/JP |
| LC279237 | Hu/GII/2016/GII.P16-GII.2/JP |
| LC279239 | Hu/GII/2016/GII.P16-GII.2/JP |
| LC279240 | Hu/GII/2016/GII.P16-GII.2/JP |
| LC279241 | Hu/GII/2016/GII.P16-GII.2/JP |
| LC279242 | Hu/GII/2016/GII.P16-GII.2/JP |
| LC279243 | Hu/GII/2016/GII.P16-GII.2/JP |
| MF167651 | Hu/GII/2017/GII.P16-GII.2/JiangSu |
| MF167652 | Hu/GII/2017/GII.P16-GII.2/JiangSu |
| KY947548 | Hu/GII/2015/GII.P16-GII.13/USA |
| MG572182 | Hu/GII/2017/GII.P16-GII.1/ShanDong |
| KY771801 | Hu/GII/2017/GII.P16-GII.2/HongKong |
