# Supplementary material for: Molecular epidemiology and spatiotemporal dynamics of norovirus associated with sporadic acute gastroenteritis during 2013–2017, Zhoushan Islands, China
Source: PLoS One. 2018 Jul 18;13(7):e0200911. doi: 10.1371/journal.pone.0200911 (PMC6051660; doi:10.1371/journal.pone.0200911)
Supplement: S2 Table — (DOC) [file pone.0200911.s002.doc]

| Strains of GII.2 VP1 used in this study. |  |
| --- | --- |
| accession numbers | sequences names |
| MH321817 | Hu/GII/GII.P16-GII.2/2017/ZhouShan |
| MH321818 | Hu/GII/GII.P16-GII.2/2017/ZhouShan |
| MH321819 | Hu/GII/GII.P16-GII.2/2017/ZhouShan |
| MH321820 | Hu/GII/GII.P16-GII.2/2017/ZhouShan |
| MH321821 | Hu/GII/GII.P16-GII.2/2017/ZhouShan |
| MH321822 | Hu/GII/GII.P16-GII.2/2017/ZhouShan |
| MH321823 | Hu/GII/GII.P16-GII.2/2017/ZhouShan |
| MH321824 | Hu/GII/GII.P16-GII.2/2017/ZhouShan |
| KY457721 | Hu/GII/GII.P16-GII.2/2014/TW |
| KY457722 | Hu/GII/GII.P16-GII.2/2014/TW |
| KY457723 | Hu/GII/GII.P16-GII.2/2014/TW |
| KY457724 | Hu/GII/GII.P16-GII.2/2015/TW |
| KY457725 | Hu/GII/GII.P16-GII.2/2015/TW |
| KY457726 | Hu/GII/GII.P16-GII.2/2016/TW |
| KY457727 | Hu/GII/GII.P16-GII.2/2016/TW |
| KY457728 | Hu/GII/GII.P16-GII.2/2016/TW |
| KY457729 | Hu/GII/GII.P16-GII.2/2016/TW |
| KY457730 | Hu/GII/GII.P16-GII.2/2016/TW |
| KY457731 | Hu/GII/GII.P16-GII.2/2016/TW |
| KY457734 | Hu/GII/GII.P16-GII.2/2016/TW |
| KY457735 | Hu/GII/GII.P16-GII.2/2016/TW |
| AB279553 | Hu/GII/GII.2/1997/JP |
| AB279554 | Hu/GII/GII.2/2001/JP |
| AB279555 | Hu/GII/GII.2/2002/JP |
| AB279556 | Hu/GII/GII.2/2002/JP |
| AB279557 | Hu/GII/GII.2/2004/JP |
| AB279558 | Hu/GII/GII.2/2004/JP |
| AB279560 | Hu/GII/GII.2/2004/JP |
| AB279561 | Hu/GII/GII.2/2004/JP |
| AB279562 | Hu/GII/GII.2/2004/JP |
| AB279563 | Hu/GII/GII.2/2004/JP |
| AB279564 | Hu/GII/GII.2/2004/JP |
| AB279565 | Hu/GII/GII.2/2004/JP |
| AB279566 | Hu/GII/GII.2/2004/JP |
| AB279567 | Hu/GII/GII.2/2004/JP |
| AB279568 | Hu/GII/GII.2/2004/JP |
| AB279569 | Hu/GII/GII.2/2005/JP |
| AB279570 | Hu/GII/GII.2/2002/JP |
| AB279571 | Hu/GII/GII.2/2004/JP |
| AB279572 | Hu/GII/GII.2/2004/JP |
| AB279573 | Hu/GII/GII.2/2004/JP |
| AB535749 | Hu/GII/GII.2/2008/JP |
| AB629941 | Hu/GII/GII.2/2010/JP |
| AB629946 | Hu/GII/GII.2/2011/JP |
| AB662850 | Hu/GII/GII.2/2005/JP |
| AB662852 | Hu/GII/GII.2/2005/JP |
| AB662853 | Hu/GII/GII.2/2005/JP |
| AB662854 | Hu/GII/GII.2/2006/JP |
| AB662856 | Hu/GII/GII.2/2006/JP |
| AB662858 | Hu/GII/GII.2/2007/JP |
| AB662859 | Hu/GII/GII.2/2008/JP |
| AB662860 | Hu/GII/GII.2/2008/JP |
| AB662861 | Hu/GII/GII.2/2008/JP |
| AB662862 | Hu/GII/GII.2/2009/JP |
| AB662863 | Hu/GII/GII.2/2006/JP |
| AB662864 | Hu/GII/GII.2/2007/JP |
| AB662865 | Hu/GII/GII.2/2007/JP |
| AB662866 | Hu/GII/GII.2/2008/JP |
| AB662867 | Hu/GII/GII.2/2008/JP |
| AB662868 | Hu/GII/GII.2/2008/JP |
| AB662869 | Hu/GII/GII.2/2008/JP |
| AB662870 | Hu/GII/GII.2/2009/JP |
| AB662871 | Hu/GII/GII.2/2009/JP |
| AB662872 | Hu/GII/GII.2/2009/JP |
| AB662873 | Hu/GII/GII.2/2009/JP |
| AB662875 | Hu/GII/GII.2/2010/JP |
| AB662876 | Hu/GII/GII.2/2010/JP |
| AB662879 | Hu/GII/GII.2/2010/JP |
| AB662880 | Hu/GII/GII.2/2010/JP |
| AB662881 | Hu/GII/GII.2/2009/JP |
| AB662884 | Hu/GII/GII.2/2009/JP |
| AB662885 | Hu/GII/GII.2/2009/JP |
| AB662886 | Hu/GII/GII.2/2009/JP |
| AB662888 | Hu/GII/GII.2/2010/JP |
| AB662889 | Hu/GII/GII.2/2010/JP |
| AB662891 | Hu/GII/GII.2/2010/JP |
| AB662892 | Hu/GII/GII.2/2010/JP |
| AB662893 | Hu/GII/GII.2/2010/JP |
| AB662895 | Hu/GII/GII.2/2010/JP |
| AB662900 | Hu/GII/GII.2/2010/JP |
| AB662901 | Hu/GII/GII.2/2010/JP |
| AY054300 | Hu/GII/GII.2/1997/USA |
| EF547398 | Hu/GII/GII.2/2000/JP |
| JQ320072 | Hu/GII/GII.2/2002/USA |
| KC464505 | Hu/GII/GII.2/2011/TW |
| KC998960 | Hu/GII/GII.2/2002/USA |
| KJ407074 | Hu/GII/GII.P16-GII.2/2011/USA |
| KT962983 | Hu/GII/GII.2/2015/TW |
| LC145786 | Hu/GII/GII.P16-GII.2/2012/JP |
| LC145787 | Hu/GII/GII.P16-GII.2/2012/JP |
| LC145788 | Hu/GII/GII.P16-GII.2/2012/JP |
| LC145789 | Hu/GII/GII.P16-GII.2/2012/JP |
| LC145790 | Hu/GII/GII.P16-GII.2/2012/JP |
| LC145791 | Hu/GII/GII.P16-GII.2/2012/JP |
| LC145792 | Hu/GII/GII.P16-GII.2/2012/JP |
| LC145793 | Hu/GII/GII.P16-GII.2/2012/JP |
| LC145794 | Hu/GII/GII.P16-GII.2/2012/JP |
| LC145795 | Hu/GII/GII.P16-GII.2/2012/JP |
| LC145796 | Hu/GII/GII.P16-GII.2/2012/JP |
| LC145797 | Hu/GII/GII.P16-GII.2/2012/JP |
| LC145798 | Hu/GII/GII.P16-GII.2/2014/JP |
| LC145799 | Hu/GII/GII.P16-GII.2/2014/JP |
| LC145800 | Hu/GII/GII.P16-GII.2/2014/JP |
| LC145801 | Hu/GII/GII.P16-GII.2/2014/JP |
| LC145802 | Hu/GII/GII.P16-GII.2/2014/JP |
| LC145803 | Hu/GII/GII.P16-GII.2/2014/JP |
| LC145804 | Hu/GII/GII.2/2014/JP |
| LC145805 | Hu/GII/GII.P16-GII.2/2014/JP |
| LC145806 | Hu/GII/GII.P16-GII.2/2014/JP |
| LC145807 | Hu/GII/GII.P16-GII.2/2014/JP |
| LC145808 | Hu/GII/GII.P16-GII.2/2014/JP |
| LC209431 | Hu/GII/GII.P16-GII.2/2013/JP |
| LC209432 | Hu/GII/GII.P16-GII.2/2012/JP |
| LC209433 | Hu/GII/GII.P16-GII.2/2012/JP |
| LC209434 | Hu/GII/GII.P16-GII.2/2014/JP |
| LC209441 | Hu/GII/GII.P16-GII.2/2014/JP |
| LC209442 | Hu/GII/GII.P16-GII.2/2013/JP |
| LC209443 | Hu/GII/GII.P16-GII.2/2013/JP |
| LC209444 | Hu/GII/GII.P16-GII.2/2013/JP |
| LC209445 | Hu/GII/GII.P16-GII.2/2012/JP |
| LC209446 | Hu/GII/GII.P16-GII.2/2012/JP |
| LC209447 | Hu/GII/GII.P16-GII.2/2011/JP |
| LC209448 | Hu/GII/GII.P16-GII.2/2011/JP |
| LC209449 | Hu/GII/GII.P16-GII.2/2011/JP |
| LC209450 | Hu/GII/GII.P16-GII.2/2014/JP |
| LC209451 | Hu/GII/GII.P16-GII.2/2011/JP |
| LC209452 | Hu/GII/GII.P16-GII.2/2011/JP |
| LC209453 | Hu/GII/GII.P16-GII.2/2011/JP |
| LC209454 | Hu/GII/GII.P16-GII.2/2010/JP |
| LC209455 | Hu/GII/GII.P16-GII.2/2013/JP |
| LC209456 | Hu/GII/GII.P16-GII.2/2013/JP |
| LC209458 | Hu/GII/GII.P16-GII.2/2014/JP |
| LC209459 | Hu/GII/GII.P16-GII.2/2010/JP |
| LC209460 | Hu/GII/GII.P16-GII.2/2010/JP |
| LC209461 | Hu/GII/GII.P16-GII.2/2009/JP |
| LC209466 | Hu/GII/GII.P16-GII.2/2012/JP |
| LC209467 | Hu/GII/GII.P16-GII.2/2011/JP |
| LC209468 | Hu/GII/GII.P16-GII.2/2011/JP |
| LC209470 | Hu/GII/GII.P16-GII.2/2014/JP |
| LC209471 | Hu/GII/GII.P16-GII.2/2011/JP |
| LC209475 | Hu/GII/GII.P16-GII.2/2013/JP |
| LC209476 | Hu/GII/GII.P16-GII.2/2013/JP |
| LC209477 | Hu/GII/GII.P16-GII.2/2013/JP |
| LC209478 | Hu/GII/GII.P16-GII.2/2012/JP |
| LC209479 | Hu/GII/GII.P16-GII.2/2011/JP |
| LC209480 | Hu/GII/GII.P16-GII.2/2010/JP |
| LC209481 | Hu/GII/GII.P16-GII.2/2010/JP |
| LC213885 | Hu/GII/GII.P16-GII.2/2015/JP |
| LC213886 | Hu/GII/GII.P16-GII.2/2016/JP |
| LC213887 | Hu/GII/GII.P16-GII.2/2016/JP |
| LC213888 | Hu/GII/GII.P16-GII.2/2016/JP |
| LC213889 | Hu/GII/GII.P16-GII.2/2016/JP |
| LC213890 | Hu/GII/GII.P16-GII.2/2016/JP |
| LC213891 | Hu/GII/GII.P16-GII.2/2016/JP |
| LC213892 | Hu/GII/GII.P16-GII.2/2016/JP |
| LC213899 | Hu/GII/GII.P16-GII.2/2016/JP |
| LC213900 | Hu/GII/GII.P16-GII.2/2016/JP |
| LC213901 | Hu/GII/GII.P16-GII.2/2016/JP |
| LC215413 | Hu/GII/GII.P16-GII.2/2016/JP |
| LC215414 | Hu/GII/GII.P16-GII.2/2016/JP |
| LC215415 | Hu/GII/GII.P16-GII.2/2016/JP |
| LC228948 | Hu/GII/GII.P16-GII.2/2014/JP |
| JN699037 | Hu/GII/GII.2/1978/MYS |
| KY421044 | Hu/GII/GII.P16-GII.2/2016/GuangDong |
| KY485115 | Hu/GII/GII.P16-GII.2/2016/GuangDong |
| KY485116 | Hu/GII/GII.P16-GII.2/2016/GuangDong |
| KY485117 | Hu/GII/GII.P16-GII.2/2016/GuangDong |
| KY485118 | Hu/GII/GII.P16-GII.2/2016/GuangDong |
| KY485119 | Hu/GII/GII.P16-GII.2/2016/GuangDong |
| KY485120 | Hu/GII/GII.P16-GII.2/2016/GuangDong |
| KY485121 | Hu/GII/GII.P16-GII.2/2016/GuangDong |
| KY485122 | Hu/GII/GII.P16-GII.2/2016/GuangDong |
| KY485123 | Hu/GII/GII.P16-GII.2/2016/GuangDong |
| KY485124 | Hu/GII/GII.P16-GII.2/2016/GuangDong |
| KY485125 | Hu/GII/GII.P16-GII.2/2016/GuangDong |
| KY485126 | Hu/GII/GII.P16-GII.2/2016/GuangDong |
| KY677828 | Hu/GII/GII.P16-GII.2/2016/HongKong |
| KY677829 | Hu/GII/GII.P16-GII.2/2016/HongKong |
| KY677830 | Hu/GII/GII.P16-GII.2/2016/HongKong |
| KY677831 | Hu/GII/GII.P16-GII.2/2016/HongKong |
| KY677832 | Hu/GII/GII.P16-GII.2/2016/HongKong |
| KY677833 | Hu/GII/GII.P16-GII.2/2016/HongKong |
| KY407217 | Hu/GII/GII.P16-GII.2/2016/GuangDong |
| KY407218 | Hu/GII/GII.P16-GII.2/2016/GuangDong |
| KY407219 | Hu/GII/GII.P16-GII.2/2016/GuangDong |
| KY407220 | Hu/GII/GII.P16-GII.2/2016/GuangDong |
| KY407221 | Hu/GII/GII.P16-GII.2/2016/GuangDong |
| KY457580 | Hu/GII/GII.P16-GII.2/2016/GuangDong |
| KY457581 | Hu/GII/GII.P16-GII.2/2016/GuangDong |
| KY457582 | Hu/GII/GII.P16-GII.2/2016/GuangDong |
| KY457583 | Hu/GII/GII.P16-GII.2/2016/GuangDong |
| KY457584 | Hu/GII/GII.P16-GII.2/2016/GuangDong |
| KY457585 | Hu/GII/GII.P16-GII.2/2016/GuangDong |
| KY457586 | Hu/GII/GII.P16-GII.2/2016/GuangDong |
| KY817742 | Hu/GII/GII.P16-GII.2/2016/HongKong |
| KY817743 | Hu/GII/GII.P16-GII.2/2016/HongKong |
| KY817744 | Hu/GII/GII.P16-GII.2/2016/HongKong |
| LC325213 | Hu/GII/GII.P16-GII.2/2016/JP |
| LC325214 | Hu/GII/GII.P16-GII.2/2016/JP |
| LC325215 | Hu/GII/GII.P16-GII.2/2016/JP |
| LC325216 | Hu/GII/GII.P16-GII.2/2016/JP |
| KY421123 | Hu/GII/GII.P16-GII.2/2016/BeiJing |
| KY421124 | Hu/GII/GII.P16-GII.2/2016/BeiJing |
| KY421125 | Hu/GII/GII.P16-GII.2/2016/BeiJing |
| KY421126 | Hu/GII/GII.P16-GII.2/2016/JiangSu |
| KY421127 | Hu/GII/GII.P16-GII.2/2016/JiangSu |
| KY421128 | Hu/GII/GII.P16-GII.2/2016/JiangSu |
| KY421129 | Hu/GII/GII.P16-GII.2/2016/GuangDong |
| KY421130 | Hu/GII/GII.P16-GII.2/2016/GuangDong |
| KY421131 | Hu/GII/GII.P16-GII.2/2016/GuangDong |
| KY421132 | Hu/GII/GII.P16-GII.2/2016/GuangDong |
| KY421133 | Hu/GII/GII.P16-GII.2/2016/GuangDong |
| KY421134 | Hu/GII/GII.P16-GII.2/2016/GuangDong |
| KY421135 | Hu/GII/GII.P16-GII.2/2016/GuangDong |
| KY421136 | Hu/GII/GII.P16-GII.2/2016/GuangDong |
| KY421137 | Hu/GII/GII.P16-GII.2/2016/GuangDong |
| KY421138 | Hu/GII/GII.P16-GII.2/2016/GuangDong |
| KY421140 | Hu/GII/GII.P16-GII.2/2016/ChongQing |
| KY421142 | Hu/GII/GII.P16-GII.2/2016/ChongQing |
| KY421143 | Hu/GII/GII.P16-GII.2/2016/ChongQing |
| AY134748 | Hu/GII/GII.2/1976/USA |
| DQ366347 | Hu/GII/GII.P22-GII.2/2004/JP |
| DQ456824 | Hu/GII/GII.P2-GII.2/2004/JP |
| JX846925 | Hu/GII/GII.P2-GII.2/1978/MYS |
| LC209435 | Hu/GII/GII.P12-GII.2/2004/JP |
| LC209436 | Hu/GII/GII.P2-GII.2/2004/JP |
| LC209437 | Hu/GII/GII.P2-GII.2/2004/JP |
| LC209438 | Hu/GII/GII.P2-GII.2/2004/JP |
| LC209439 | Hu/GII/GII.Pe-GII.2/2014/JP |
| LC209440 | Hu/GII/GII.P2-GII.2/2015/JP |
| LC209457 | Hu/GII/GII.P2-GII.2/2015/JP |
| LC209462 | Hu/GII/GII.P2-GII.2/2006/JP |
| LC209463 | Hu/GII/GII.P2-GII.2/2008/JP |
| LC209464 | Hu/GII/GII.P2-GII.2/2004/JP |
| LC209465 | Hu/GII/GII.P2-GII.2/2010/JP |
| LC209469 | Hu/GII/GII.P2-GII.2/2014/JP |
| LC209472 | Hu/GII/GII.P2-GII.2/2010/JP |
| LC209473 | Hu/GII/GII.P2-GII.2/2010/JP |
| LC209474 | Hu/GII/GII.P2-GII.2/2010/JP |
| KY421144 | Hu/GII/GII.P16-GII.2/2016/ChongQing |
| KY421145 | Hu/GII/GII.P16-GII.2/2016/ChongQing |
| KY421146 | Hu/GII/GII.P16-GII.2/2016/ChongQing |
| KY421147 | Hu/GII/GII.P16-GII.2/2016/ChongQing |
| KY421148 | Hu/GII/GII.P16-GII.2/2016/ChongQing |
| KY421149 | Hu/GII/GII.P16-GII.2/2016/ChongQing |
| KY421150 | Hu/GII/GII.P16-GII.2/2016/JiangSu |
| KY421151 | Hu/GII/GII.P16-GII.2/2016/BeiJing |
| KY421152 | Hu/GII/GII.P16-GII.2/2016/FuJian |
| KY421153 | Hu/GII/GII.P16-GII.2/2016/FuJian |
| KY421154 | Hu/GII/GII.P16-GII.2/2016/BeiJing |
| KY421155 | Hu/GII/GII.P16-GII.2/2016/GuangDong |
| KY421156 | Hu/GII/GII.P16-GII.2/2016/GuangDong |
| KY421157 | Hu/GII/GII.P16-GII.2/2016/GuangDong |
| AB662883 | Hu/GII/GII.P16-GII.2/2009/JP |
| KY806290 | Hu/GII/GII.P16-GII.2/2017/JiangSu |
| KY806291 | Hu/GII/GII.P16-GII.2/2017/JiangSu |
| LC279238 | Hu/GII/GII.P16-GII.2/2016/JP |
| KY817745 | Hu/GII/GII.P16-GII.2/2016/HongKong |
| KY817746 | Hu/GII/GII.P16-GII.2/2016/HongKong |
| KY817747 | Hu/GII/GII.P16-GII.2/2016/HongKong |
| KY817748 | Hu/GII/GII.P16-GII.2/2017/HongKong |
| KY817749 | Hu/GII/GII.P16-GII.2/2017/HongKong |
| KY817750 | Hu/GII/GII.P16-GII.2/2017/HongKong |
| KY817751 | Hu/GII/GII.P16-GII.2/2017/HongKong |
| KY817752 | Hu/GII/GII.P16-GII.2/2017/HongKong |
| KY771081 | Hu/GII/GII.P16-GII.2/2017/HongKong |
| KY806292 | Hu/GII/GII.P16-GII.2/2017/JiangSu |
| KY806293 | Hu/GII/GII.P16-GII.2/2017/JiangSu |
| KY806294 | Hu/GII/GII.P16-GII.2/2017/JiangSu |
| KY806295 | Hu/GII/GII.P16-GII.2/2017/JiangSu |
| KY806300 | Hu/GII/GII.P16-GII.2/2017/JiangSu |
| KY806301 | Hu/GII/GII.P16-GII.2/2017/JiangSu |
| LC279234 | Hu/GII/GII.P16-GII.2/2016/JP |
| LC279242 | Hu/GII/GII.P16-GII.2/2016/JP |
| LC279243 | Hu/GII/GII.P16-GII.2/2016/JP |
| MF167651 | Hu/GII/GII.P16-GII.2/2017/JiangSu |
| MF167652 | Hu/GII/GII.P16-GII.2/2017/JiangSu |
