# Supplementary material for: Molecular epidemiology and spatiotemporal dynamics of norovirus associated with sporadic acute gastroenteritis during 2013–2017, Zhoushan Islands, China
Source: PLoS One. 2018 Jul 18;13(7):e0200911. doi: 10.1371/journal.pone.0200911 (PMC6051660; doi:10.1371/journal.pone.0200911)
Supplement: S3 Table — (DOC) [file pone.0200911.s003.doc]

S3. The primers sequences used for amplifying complete GII.P16-GII.2 strain genome

| Primers | Sequences |
| --- | --- |
| GII.P16-GII.2-1F^a^ | GTGAATGAAGATGGCGTCTAAC |
| GII.P16-GII.2-669R | AGCCATGAGTCATTGAGGTCT |
| GII.P16-GII.2-514F | GTTGTCATTGTACTGGCGTGT |
| GII.P16-GII.2-976R | GGAGCGATCATAGCAGACACAT |
| GII.P16-GII.2-791F | TTCATTGTCCACATTATTCACCAG |
| GII.P16-GII.2-1269R | GGTTATTTTCTATTGCCTCAAGGTCTA |
| GII.P16-GII.2-1097F | GTCCTCAGCAGCATCAACACTC |
| GII.P16-GII.2-1882R | CTTTTCTACTTCTGGTGCCTCA |
| GII.P16-GII.2-1674F | ATGCTCTTCGCATACAAGAATT |
| GII.P16-GII.2-2121R | TGAAGGTGGTGATTGTGGGAC |
| GII.P16-GII.2-2005F | ATGGGAAAGGAGTGATGAAGACC |
| GII.P16-GII.2-2473R | CCGGCAATTTGTATGATGGA |
| GII.P16-GII.2-2338F | TAGAAAAGGTCAAGAACACCTCTGT |
| GII.P16-GII.2-2932R | TTCTGAACCTGTTACTAGCCCTA |
| GII.P16-GII.2-2786F | TACTATGAGGAGCTCGCCATT |
| GII.P16-GII.2-4058R | CTGTGTACACTGGTGTCATGTGTT |
| GII.P16-GII.2-3875F | CAAAAATGGACATACGCACAG |
| GII.P16-GII.2-5259R | CCTGGACAAAATTTGCTCTAA |
| GII.P16-GII.2-5025F | TGAGGTTTTCTGACTTGAGCA |
| GII.P16-GII.2-6731R | CTACAAAAGCTCCAGCCATTA |
| GII.P16-GII.2-6621F | GCCAACGGGTACTTCAGATT |
| GII.P16-GII.2-7536R | AAAAGGTAAAATCAATTCATCCTAA |

^a^The nucleotide position was according to KY421122/CHN/2016/Jiangsu strain
